# Realization of a 27.5 °C Atmospheric Microwave Plasma Jet at 8 W for Surface Modification of Thermosensitive Polymers

**DOI:** 10.3390/polym17162183

**Published:** 2025-08-09

**Authors:** Dongxue Han, Wencong Zhang, Yong Yang, Yuantao Huang, Jiangqi Yu, Li Wu, Wenyan Tian, Huacheng Zhu

**Affiliations:** 1 School of Electronics and Information Engineering, Guiyang University, Guiyang 550005, China; handongxue0609@outlook.com (D.H.); yangyong1933@outlook.com (Y.Y.); huangyuantao2001@126.com (Y.H.); yjq0301@outlook.com (J.Y.); 2 IAEM (Institute of Applied Electro Magnetics), College of Electronics and Information Engineering, Sichuan University, Chengdu 610065, China; wuli1307@scu.edu.cn (L.W.);; 3 School of Electronic Information Engineering, Taiyuan University of Science and Technology, Taiyuan 030024, China

**Keywords:** atmospheric pressure, plasma jets, microwave plasma jet, surface modification

## Abstract

Atmospheric pressure plasma jets (APPJs) enable efficient solvent-free surface modification to enhance the wettability, adhesion, and biocompatibility of polymers. However, conventional APPJs often generate high temperatures and energetic particles, which lead to melting, surface degradation, and molecular damage of thermosensitive polymers, thus limiting their scope of application. This study demonstrates an optimized atmospheric pressure microwave plasma jet (MPJ) operating at 8 W microwave power, achieving gas temperatures as low as 27.5 °C—only 2 °C above ambient. Direct skin contacts with the plasma jet for 150 s resulted in a maximum temperature of 35 °C without discomfort. In addition, the MPJ significantly enhances the surface hydrophilicity of TPU, PVC, and POM materials without causing damage. The designed MPJ has low gas temperature and good discharge stability, providing a new solution for plasma surface modification of thermosensitive materials.

## 1. Introduction

Atmospheric pressure plasma jets (APPJs) enable efficient solvent-free surface modification of polymers under ambient conditions [1,2]. By generating electrons, ions, radicals (e.g., O, OH, NO), and excited metastable states [3,4,5], APPJs can alter the surface chemistry and morphology of materials, thereby enhancing wettability, adhesion, and biocompatibility [6,7,8,9]. However, conventional systems such as DBD discharges, DC discharges, and gliding arc discharges typically produce high temperatures and highly energetic species [10,11,12]. When used on thermosensitive polymers—such as biodegradable polyesters (e.g., PCL, PGA, PLLA) and functional hydrogels—these conventional jets often cause thermal damage. Such damage may manifest as local melting, surface degradation, chain scission, or unwanted cross-linking. In contrast, microwave plasma jets (MPJs) combine electrodeless operation, high electron density, abundant reactive species, and reduced thermal effects [13,14,15]. This enables efficient surface treatment while minimizing damage, particularly on temperature-sensitive materials.

Nevertheless, under atmospheric pressure, the high ionization threshold necessitates elevated microwave power to sustain plasma discharge, which inevitably results in heat accumulation and increased plasma temperatures [16,17]. Consequently, generating microwave plasma plumes near ambient temperature remains a significant challenge. Commonly employed microwave plasma jet configurations include designs based on rectangular waveguides [18,19,20], microstrip lines [21,22,23,24], and coaxial transmission lines [25,26]. Among these, rectangular waveguide structures are favored for their simplicity but typically require higher power levels, leading to elevated plume temperatures. In our previous design, a surface wave jet based on a rectangular waveguide achieved a plume tail temperature as low as 22 °C at 25 W [27]. However, the limited plasma length restricted its applicability for practical polymer surface treatment. Microstrip line-based jets can produce plume temperatures ranging from 30 °C to 65 °C [28,29,30], suitable for low thermal impact processing. However, their structural complexity and susceptibility to long-term thermal damage—such as dielectric substrate melting or degradation—pose limitations. In contrast, coaxial transmission line-based atmospheric pressure MPJs can generate strong electric fields at low power levels, enabling low-temperature plasma jets with tail gas temperatures controllable within 30–60 °C [31,32,33,34,35,36,37,38,39,40,41]. Therefore, the coaxial transmission line configuration has been demonstrated as one of the effective approaches for generating atmospheric pressure low-temperature plasma jets, exhibiting significant potential for applications in surface modification.

Although coaxial MPJs have the potential for low-temperature operation, improper nozzle structure design can easily lead to discharge instability, ignition difficulties, or internal conductor ablation. Therefore, achieving stable and ultra-low-temperature (<30 °C) plasma jets at very low power levels (<10 W) still poses challenges. For instance, the MPJ structures proposed in [33,40,41] enhance the electric field intensity at the discharge end by narrowing the discharge gap and sharpening the tip of the inner electrode. This configuration simultaneously improves local convective heat dissipation, effectively maintaining the gas temperature at a relatively low level. However, it should be noted that excessively small gaps or highly restricted gas flow channels can increase local gas velocity, potentially leading to jet instability or premature plasma extinction. Moreover, under prolonged exposure to strong electric fields, the sharpened tip electrode faces a significant risk of erosion, compromising device longevity and repeatability. Conversely, uncompressed nozzle structures, as employed in references [32,35,39], demonstrate improved discharge stability. However, they exhibit limited convective and diffusive cooling effects, resulting in elevated jet temperatures that may not satisfy the low-temperature requirements for treating thermosensitive polymers. Therefore, achieving an optimal balance between electric field enhancement, thermal management, and discharge stability is crucial in the design of atmospheric pressure MPJs for realizing stable low-temperature discharges.

To overcome these challenges, this study proposes a structurally optimized atmospheric pressure MPJ device, building upon our previous research that focused on the design of MPJs. Through adjustments to the nozzle structure, specifically the discharge gap dimensions and the morphology of the inner electrode tip, the device achieves stable low-temperature plasma jet generation under low power conditions. This provides crucial technical support for the thermal damage-free processing of heat-sensitive materials. To validate the application potential of this device for surface modification of heat-sensitive polymers, an experimental platform utilizing argon as the working gas was established. The discharge characteristics and gas temperature distribution of the MPJ were systematically investigated under varying microwave power levels and gas flow rates. Experimental results demonstrated that, at a microwave input power of 8 W, the gas temperature measured in the plasma jet tail region was as low as 27.5 °C, confirming the excellent low-temperature discharge performance of the device. Furthermore, three representative thermosensitive polymers—thermoplastic polyurethane (TPU), polyvinyl chloride (PVC), and polyoxymethylene (POM)—were selected for static water contact angle measurements and surface temperature analysis following plasma treatment. The results revealed a significant enhancement in the surface hydrophilicity of all polymers following MPJ treatment. Crucially, the surface temperature during processing consistently remained below 50 °C, effectively avoiding the risk of thermal damage. In summary, the MPJ system proposed offers a promising low-temperature plasma solution for nondestructive surface modification of thermosensitive polymeric materials.

## 2. Methods 

### 2.1. Numerical Design

**Figure 1:**
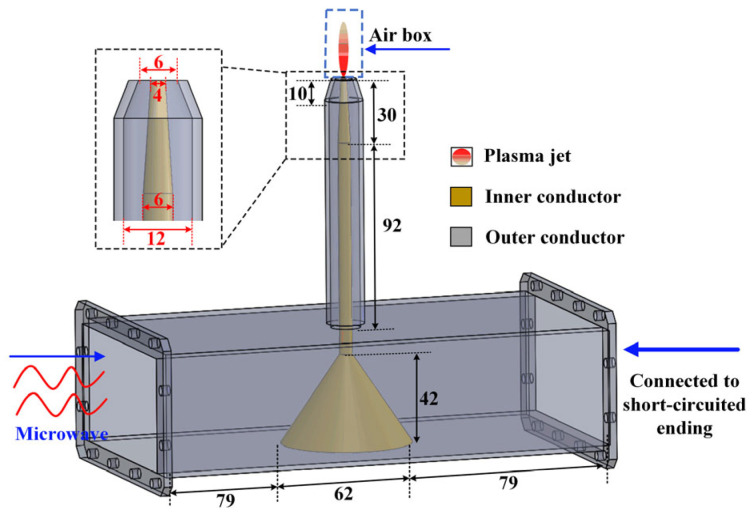
Schematic graph of the MPJ device structure (unit: mm).

Figure 1 depicts the structure and dimensions of the optimized MPJ operating at 2.45 GHz. The device is based on a standard WR340 waveguide design. Both ends of the waveguide feature an open structure: one end serves as the microwave feed port where a wave port boundary condition is applied for excitation, while the other end is connected to a movable short-circuited ending plunger. To enhance microwave transmission efficiency, a tapered transition design is employed for the waveguide-to-coaxial conversion. The coaxial nozzle features a conical open-ended structure, with its geometry specifically engineered to balance impedance matching and electric field focusing. The propagation and dissipation characteristics of electromagnetic waves within the MPJ are governed by the Helmholtz equation, as formulated in Equation (1). Where $\vec{E}$ represents the microwave electric field. The constants $\varepsilon_{0}=8.854\times 10^{-12}\;{F\cdot m}^{-1}$,  ω , and $k_{0}$ denote the vacuum permittivity, the microwave angular frequency, and the free-space wavenumber, respectively. $\varepsilon_{r}$, $\mu_{r}$, and σ are the relative permittivity, the relative permeability, and electric conductivity of the corresponding medium, respectively. Furthermore, an air column with an open boundary condition is incorporated at the nozzle outlet to accurately simulate microwave radiation characteristics. All other boundaries are defined as perfect electric conductors (PECs) to eliminate boundary effects. Notably, the waveguide-to-coaxial transition of the designed MPJ was introduced primarily to accommodate experimental requirements for monitoring incident and reflected power. In practical applications, the rectangular waveguide can be eliminated, allowing direct coaxial connection to the microwave source for a more compact and simplified system.

(1) $$\nabla \times \left(\mu_{r}^{-1}\nabla \times \vec{E}\right)-k_{0}^{2}\left(\varepsilon_{r}-\frac{j\sigma }{\omega \varepsilon_{0}}\right)\vec{E}=0$$

(2) $$\varepsilon_{rp}=1-\frac{n_{e}q^{2}}{{m_{e\;}\varepsilon_{0}(\omega }^{2}+{V_{m}}^{2})}-j\frac{n_{e}q^{2}V_{m}}{{m_{e\;}\varepsilon_{0}\omega (\omega }^{2}+{V_{m}}^{2})}$$

The plasma plume can be regarded as a dielectric medium with the relative permittivity $\varepsilon_{rp}\;$, as given in Equation (2). Where $\nu_{m}$ denotes the electron–neutral elastic collision frequency, while $\omega_{p}$ represents the plasma frequency. The fundamental plasma properties are determined by the electron number density $n_{e}$, the electron mass $m_{e}$, and the elementary charge q. The $n_{e}$ and $\nu_{m}$ values in actual scenarios exhibit spatially non-uniform distributions within the plasma jet, which can be calculated using Multiphysics models. Such models can accurately characterize the coupling of physical processes in microwave plasmas and thereby facilitate insights into discharge mechanisms. However, the highly coupled nature of these processes renders fully three-dimensional models that are computationally intensive and resource-demanding [42]. Consequently, to enhance modeling efficiency, the Drude model is generally employed as an effective approximation especially in the design of microwave sources [43]. However, the validity of this model critically depends on the assumptions regarding key plasma parameters, specifically electron number density and electron collision frequency. In this study, an electron number density is set to $n_{e}=1\times 10^{20}m^{-3}$ and a collision frequency to $\nu_{m}=1\times 10^{10}\;Hz$. These values align with those reported in previous numerical simulations and experimental studies [27,43,44].

**Figure 2:**
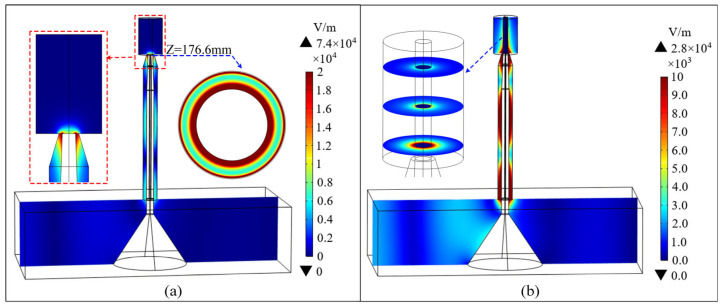
Distributions of the microwave electric field: (**a**) without the plasma column; (**b**) with the plasma column.

To investigate the electric field distribution within the designed MPJ and the propagation behavior of electromagnetic waves in the presence of plasma, Figure 2 illustrates the microwave electric fields in the MPJ system with and without the presence of a plasma column. These simulations were performed using the commercial finite element software COMSOL Multiphysics 6.3. The computational model without plasma comprised a total of 1,424,748 mesh elements and a complete calculation required approximately 10 min. When considering the plasma column based on the Drude model, the total mesh elements enhanced to 1,821,880 and its computational time increased to approximately 20 min. All simulations were conducted on a computer equipped with an Intel i7-10700K processor and 128 GB of RAM. As shown in Figure 2a, the electric field reaches its peak intensity at the tip of the nozzle, where the localized high-field region enhances gas breakdown efficiency and effectively triggers the discharge process. It is noteworthy that, once gas discharge occurs, the resulting plasma behaves as a dynamic load that can absorb, reflect, and even shield electromagnetic waves. If the structural design is inappropriate, such as an excessively small discharge gap, after triggering the gas discharge, the coupled effects of the electromagnetic field distribution and gas flow near the nozzle may confine the plasma within the discharge gap, manifesting as fan-shaped or rotating discharges [45], thereby hindering the formation of an outward-propagating plasma jet.

Therefore, to investigate the formation mechanisms of post-discharge plasma jets, the Drude model is employed to simulate the propagation characteristics of electromagnetic waves following discharge. Figure 2b shows the simulation results based on the Drude model. As shown, the electromagnetic waves propagate along the surface of the plasma column and gradually dissipate. This behavior is attributed to the excitation of a surface wave mode at the interface when microwaves propagate between the plasma with a negative dielectric constant and the surrounding air with a positive dielectric constant [46]. In this mode, microwaves propagate efficiently along the plasma column, continuously supplying energy to sustain the plasma. Consequently, stable plasma maintenance is achieved even at a relatively low input power.

### 2.2. Experimental Measurement

**Figure 3:**
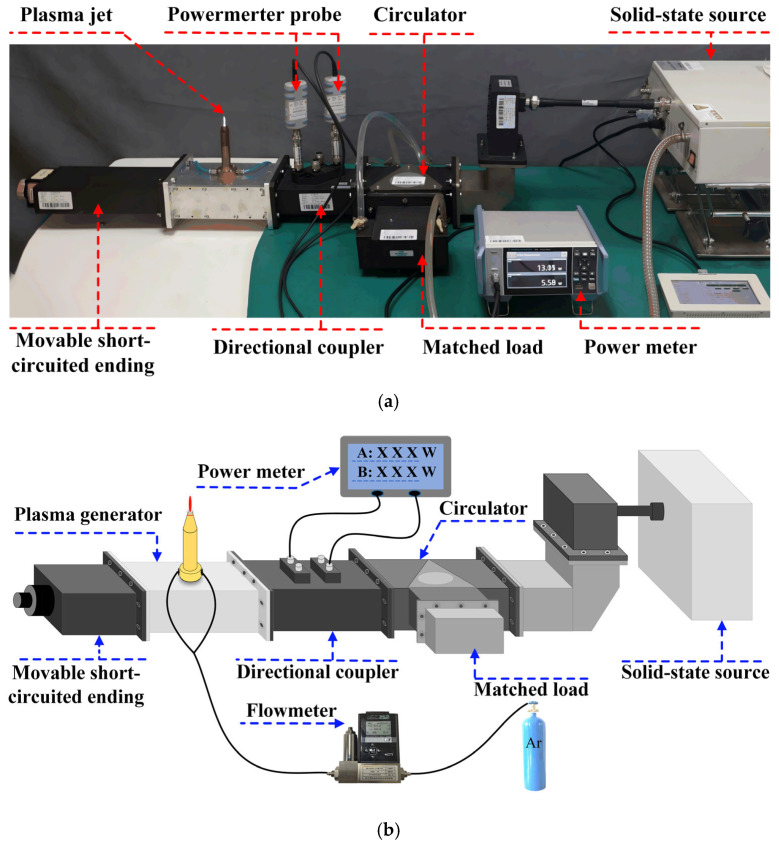
(**a**) Photograph of the experimental system; (**b**) diagram of the experimental system.

Figure 3 presents the photographic image and schematic diagram of the MPJ experimental setup developed. The system primarily consists of a solid-state microwave generator (WSPS-2450-1K-CCWA; Chengdu Wattsine Electronic Technology Co., Ltd., Chengdu, Sichuan, China; ±1 W accuracy), a circulator, a bidirectional coupler, a water-cooled matched load, a microwave power meter (ROHDE & SCHWARZ, NRX; Rohde &; Schwarz GmbH &; Co. KG, Munich, Germany; ±0.01 W accuracy), and an MPJ device based on a standard WR430 rectangular waveguide. The microwave power is supplied by a solid-state generator operating at 2.45 GHz, with an output power continuously adjustable from 0 to 1000 W. A three-port circulator connects the microwave source to both the bidirectional coupler and the matched load, serving to prevent reflected power from damaging the source. The bidirectional coupler and the power meter enable precise measurement of both the incident and the reflected microwave powers to the MPJ, thereby facilitating evaluation of the system’s power efficiency.

In this study, gas discharge is triggered by a sharpened copper conductor that enhances the local electric field at the nozzle tip and causes microwave gas breakdown. The morphology of the plasma jet plume is captured using a high-resolution digital camera (Canon EOS R10; Canon (China) Co., Ltd., Beijing, China). Typically, the optical emission spectrum of the plasma can be collected using a high-precision spectrometer to estimate the gas temperature. However, under atmospheric pressure conditions, the plasma typically exhibits a strongly non-equilibrium state, and its high particle density tends to induce self-absorption effects, resulting in distorted spectral line profiles. In addition, the millimeter-scale structure of the plasma jet demands high spatial resolution, which further limits the accuracy and applicability of optical emission spectroscopy for spatially resolved temperature diagnostics within the jet plume.

To obtain more reliable temperature data, this study further employs an infrared thermal imaging camera (TESTO 885; Testo SE & Co. KGaA, Lenzkirch, Germany; ±0.03 °C accuracy) and a thermocouple sensor (DT1311; Wenzhou Hanbon Electronics Co., Ltd., Wenzhou, Zhejiang, China; ±0.01 °C accuracy) to characterize the gas temperature at the tip of the plasma plume. Specifically, the thermocouple probe is positioned approximately 3 mm downstream along the central axis of the plume for in situ contact temperature measurement. During thermal imaging, the distance between the IR camera and the plasma source is fixed at 30 cm to ensure image consistency and accuracy. High-purity argon (99.999%) is used as the working gas and is symmetrically introduced into the MPJ system from both sides of the coaxial structure. The gas flow rates are set to 10 SLM, 15 SLM, and 20 SLM, representing three typical operational conditions corresponding to low, medium, and high flow regimes. The flow rate is precisely controlled and monitored using a mass flow controller (SLD-MFC; Nanjing Senlod Automation equipment Co., Ltd., Nanjing, China; ±0.002 F·S accuracy) with an adjustable range of 0–30 SLM. All experiments are conducted in Guiyang between 09:00 and 12:00 on 22 June 2025 under standard atmospheric pressure (101.325 kPa) and ambient temperature (25.5 °C). Each experimental set is repeated three times to ensure measurement reliability and reproducibility.

## 3. Results and Discussion

**Figure 4:**
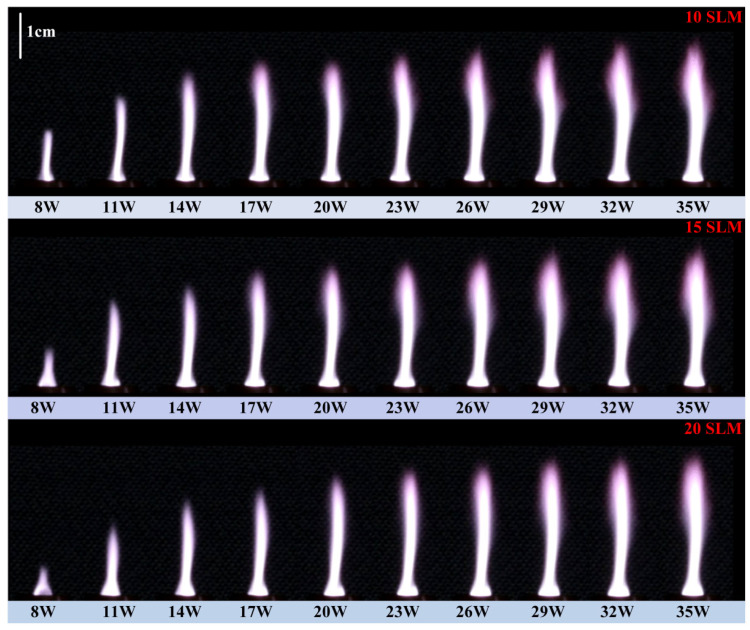
Photographs of gas discharge corresponding to different gas flow rates under different microwave input powers.

Figure 4 displays the plasma jet images generated by the proposed MPJ system under varying microwave powers and argon flow rates, while Table 1 quantitatively summarizes the corresponding jet lengths. The experimental results indicate that stable plasma jets can be sustained within an input power range of 8–35 W, further validating the structural rationality of the MPJ design based on the Drude model. As shown in Figure 4 and Table 1, the plasma jet brightness, axial length, and volumetric discharge region increase significantly with rising input power. Table 1 further provides a quantitative assessment of jet length variations under different argon flow rates, showing that jet length increases with higher input power but gradually saturates in the high-power regime. This trend can be primarily attributed to the enhanced electric field strength at elevated powers, which allows electromagnetic waves to propagate farther along the plasma column, thereby sustaining a longer plasma channel.

Conversely, as the gas flow rate increases, the jet length tends to decrease slightly, and the luminous region becomes more confined. This phenomenon is likely due to the reduced residence time of reactive species within the discharge region under high flow velocity conditions, which limits the continuity of axial discharges. In addition, the increase in gas flow promotes lateral diffusion of the jet, which contributes to a more uniform spatial distribution of the plasma plume. It is worth noting that the evolution of jet temperature under different combinations of microwave power and gas flow rate remains unclear and requires further investigation through dedicated measurements and energy transport analysis.

**Figure 5:**
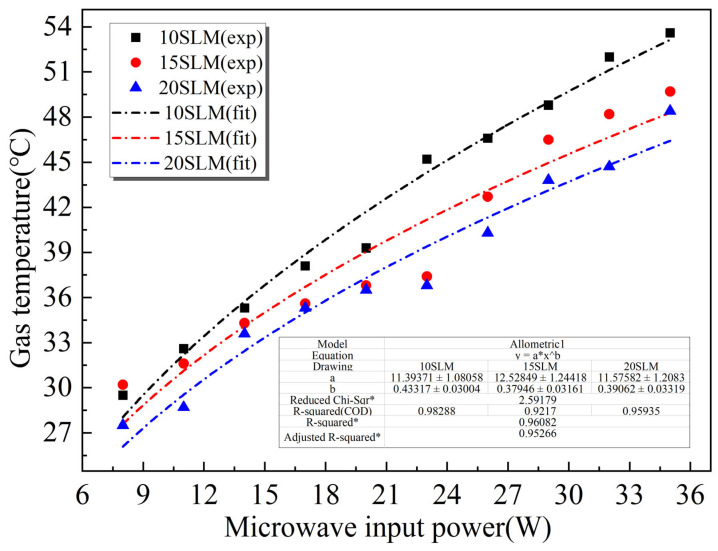
Gas temperature in the jet tail for different gas flow rates at different microwave input powers. (Note: The physical quantities marked with * in the table are indicators of the fitting effect. The closer the value is to 1, the better the fitting effect).

Figure 5 illustrates the variation of gas temperature in the plasma jet tail region under different microwave input powers (8–35W) with corresponding gas flow rates of 10, 15, and 20 SLM. Experimental results (indicated by symbols) demonstrate that, at the minimum input power (8 W), the gas temperature reaches as low as 27.5 °C, closely approaching ambient levels. While increasing microwave power induces a gradual temperature elevation in the jet tail region, the temperature remains below 56 °C throughout the operational power range (8–35 W), confirming the superior low-temperature characteristics of the designed MPJ device.

To further quantify the relationship between input power and temperature rise, a power–law function $T=a\times P^{b}$ was used to fit the experimental data, where T represents the gas temperature and P denotes the microwave input power. The dashed lines in the figure represent the fitted curves. For all flow rates, the fitted curves show strong agreement with the experimental data, with coefficients of determination ($R^{2}$) exceeding 0.9, indicating a high quality of fit. The corresponding fitting parameters are listed in the embedded table in the figure. The results indicate that, under identical microwave power conditions, higher gas flow rates correspond to lower temperature rises. This phenomenon is primarily attributed to the enhanced heat-carrying capacity of the gas at increased flow rates, which promotes more efficient convective heat transfer, thus effectively suppressing the temperature increase. In contrast, under constant flow rate conditions, microwave power serves as the dominant energy input source; its augmentation significantly enhances energy deposition within the gas, thereby leading to a rise in gas temperature. Moreover, compared with gas flow rate, microwave power plays a more dominant role in determining the thermal characteristics of the plasma jet. This is attributed to the enhanced electric field and increased electron energy induced by higher power, which lead to a significantly greater energy deposition in the gas.

In the processing of thermally sensitive biomedical materials like biodegradable polyesters (e.g., PCL, PGA, PLLA) and functional hydrogels, temperatures exceeding 40 °C can induce thermal degradation, cross-linking, softening, or deformation [47,48,49]. Therefore, the working gas temperature of the plasma jet must be strictly controlled below the material’s thermal decomposition threshold, ideally approaching room temperature, to preserve structural and mechanical integrity. Consequently, the developed atmospheric pressure MPJ device, with its outstanding low-temperature characteristics and excellent tunability, exhibits broad applicability and potential for the surface modification treatment of thermosensitive polymeric materials.

**Figure 6:**
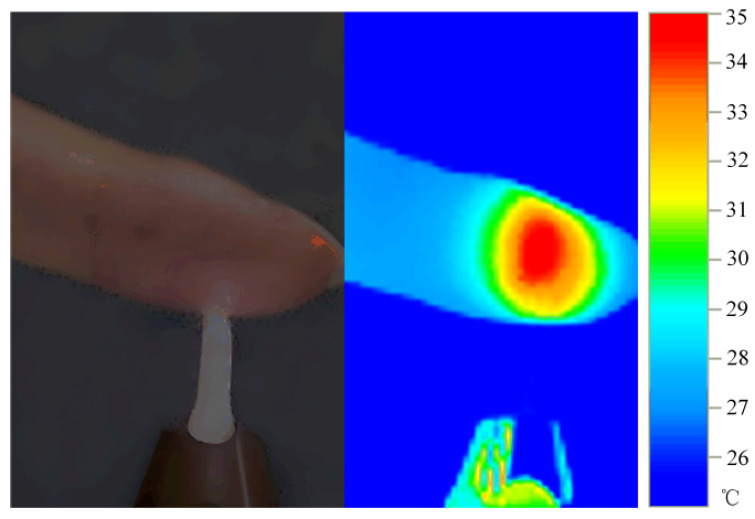
Photograph and thermal image of argon plasma jet in contact with human skin (microwave input power: 11 W; gas flow rate: 20 SLM).

Figure 6 displays the visible light image and corresponding infrared thermogram of a human fingertip after approximately 150 s of direct exposure to an argon plasma jet. The thermal distribution clearly indicates that the plasma jet induces a highly localized thermal effect restricted to the area of direct skin contact, with no evident thermal conduction to surrounding tissues. Remarkably, despite prolonged contact, the surface temperature remained around 35 °C without eliciting thermal pain, confirming the plasma’s stable low-temperature behavior under atmospheric conditions. This sustained low-temperature operation enables safe surface modification of thermosensitive polymers, effectively avoiding heat-induced deformation, softening, degradation, or performance loss. Additionally, the continuous treatment mode enhances the diffusion and interfacial reactivity of active species (e.g., oxygen-containing radicals), addressing the limitations of pulsed low-temperature treatments regarding modification depth and surface uniformity. These findings provide robust support for controlled and efficient polymer surface functionalization via low-temperature plasmas.

**Figure 7:**
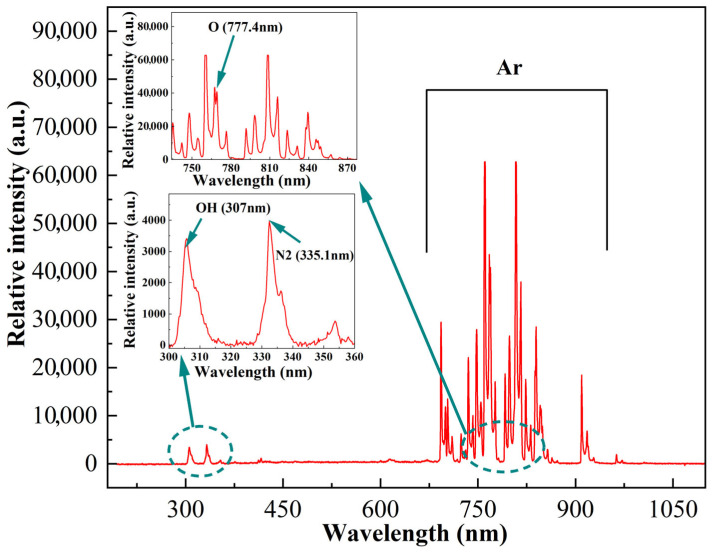
Emission spectrum of the MPJ with the working gas of argon.

Figure 7 shows the emission spectrum of an argon plasma generated at an input power of 14 W with an argon gas flow rate of 20 SLM. The spectrum was acquired using a spectrometer (ER4000EX; Shanghai choptics Instrument Equipment Co., Ltd., Shanghai, China.) with a resolution ranging from 0.1 to 4.88 nm. The optical probe was positioned 4 cm from the nozzle exit, vertically aligned with the nozzle axis, and 2.5 cm horizontally from the center of the plasma plume. Spectral analysis indicates that the emission spectrum at this location is dominated by spectral lines corresponding to various energy levels of atomic argon. Simultaneously, a Vegard–Kaplan system was detected at 335.1 nm ($A^{3}\sum_{u}^{+}-X^{1}\sum_{g}^{+})$ and N_2_ was detected at 335.1 nm [50]. Notably, characteristic peaks of highly reactive radicals were observed, including the highly reactive radicals like OH (2S → 2P) at 307 nm [51] and excited atomic oxygen (O, 3p^5^P → 3s^5^S) at 777.4 nm [52]. These strong oxidizing active substances have significant application potential in the fields of plasma–surface interaction and material surface modification.

**Figure 8:**
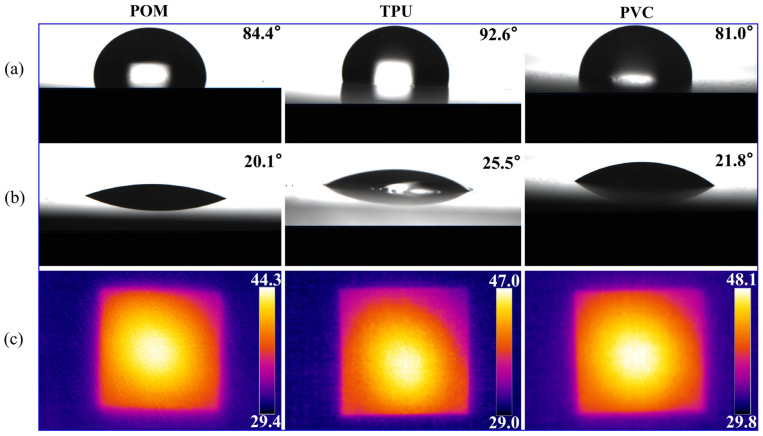
Surface modification of polymers using proposed MPJ (microwave input power: 14 W; gas flow rate: 20 SLM): (**a**) static water contact angles (WCA) before MPJ treatment; (**b**) static water contact angles after 60 s MPJ treatment; (**c**) surface temperature distribution (°C) measured of materials after 60 s MPJ treatment.

To evaluate the applicability and effectiveness of the proposed MPJ device for surface modification of heat-sensitive polymers, this study selected three representative thermosensitive polymeric materials as research subjects: polyoxymethylene (POM), thermoplastic polyurethane (TPU), and polyvinyl chloride (PVC). The investigation focused on assessing the changes in static water contact angle (WCA) and the surface temperature distribution of the materials following 60 s of MPJ treatment, as illustrated in Figure 8. Figure 8a,b display representative static WCA images for each material before and after treatment, respectively. The results demonstrate that the contact angles of all three polymers decreased significantly after MPJ treatment, indicating a substantial enhancement in surface hydrophilicity. Specifically, the contact angle of POM decreased from 84.4° to 20.1°, TPU from 92.6° to 25.5°, and PVC from 81.0° to 21.8°. These findings confirm the capability of the MPJ system to modulate the surface energy across diverse polymers. Figure 8c presents infrared thermographic images of the samples after treatment, characterizing their surface temperature distribution. The data reveal that the maximum surface temperature for all materials remained below 50 °C after 60 s of MPJ exposure, with recorded maximum temperatures of 44.3 °C for POM, 47.0 °C for TPU, and 48.1 °C for PVC. This result further substantiates the pronounced low-temperature discharge characteristics of the proposed MPJ technology, confirming its applicability for the surface modification of heat-sensitive materials.

## 4. Conclusions

This study designed an optimized atmospheric pressure microwave plasma jet. The device can generate a high-intensity microwave electric field under ultra-low power conditions, enabling stable excitation of low-temperature plasma at atmospheric pressure, suitable for surface treatment of thermally sensitive materials. Subsequently, an experimental platform was established to systematically validate the device’s performance. The main conclusions are as follows:

(1) The designed MPJ system produces an argon plasma jet with a minimum gas temperature of 27.5 °C at an input power as low as 8 W, which is only approximately 2 °C above ambient temperature.
(2) During a 150 s direct skin contact test, the maximum surface temperature remained around 35 °C without causing thermal discomfort or pain, demonstrating excellent low-temperature performance suitable for long-term treatment of heat-sensitive materials.
(3) Within the microwave power range of 8–35 W and gas flow rates of 10–20 SLM, the gas temperature consistently remained below 56 °C, further confirming the device’s low-temperature adaptability and thermal stability.
(4) The plasma gas temperature increased with rising microwave input power and decreased with increasing gas flow rate. Among these factors, microwave power exerted the most significant influence on temperature variation.
(5) Static water contact angles of POM, TPU, and PVC polymer materials significantly decreased after 60 s of MPJ treatment, demonstrating substantially enhanced surface hydrophilicity. Additionally, all material surfaces were maintained below 50 °C post-treatment.

In summary, the low-temperature MPJ designed in this study offers a practical and efficient technical approach for the surface modification of thermally sensitive polymers. Notably, the MPJ proposed in this study allows for the omission of the rectangular waveguide structure without compromising its overall performance. This design significantly enhances the applicability and operational convenience for polymer material processing. However, due to the limited treatment area of the current device, its applicability in large-area surface modification or high-throughput scenarios remains constrained. Future work will focus on optimizing the uniformity and spatial coverage of the plasma plume and exploring its application to various polymer materials, thereby broadening its practical value and industrial potential.

## Figures and Tables

**Table 1:** Plasma jet length(mm) at different gas flow rates under different microwave powers.

Pin (W)	10 L/min	15 L/min	20 L/min
8	11	9	7
11	19	19	17
14	24	23	21
17	26	25	23
20	27	28	27
23	29	27	28
26	30	29	29
29	31	30	30
32	31	30	31
35	32	30	31

## Data Availability

Data are contained within this article.
